# Case Report: Preservation of Otolithic Function After Triple Semicircular Canal Occlusion in a Patient With Intractable Ménière Disease

**DOI:** 10.3389/fneur.2021.713275

**Published:** 2021-12-23

**Authors:** Yuzhong Zhang, Ying Cheng, Zichen Chen, Feiyun Chen, Qing Zhang

**Affiliations:** ^1^Department of Otorhinolaryngology-Head and Neck Surgery, The Second Affiliated Hospital of Xi'an Jiaotong University, Xi'an, China; ^2^Department of Otorhinolaryngology-Head and Neck Surgery, Xinhua Hospital, Shanghai Jiaotong University School of Medicine, Shanghai, China

**Keywords:** Ménière disease, triple semicircular canal occlusion, vestibular evoked myogenic potentials, vestibular function preservation, hearing preservation

## Abstract

Operative measures are considered when medical treatment fails to control vertigo in patients with intractable Ménière disease. The present report discusses a case in which triple semicircular canal occlusion was performed in a 30-year-old female patient who responded poorly to previously performed endolymphatic sac surgery. Her vestibular and auditory functions were evaluated both before and after surgery. Class A control of vertigo was achieved during the 76-month postoperative follow-up period. Ocular and cervical vestibular evoked myogenic potentials could be elicited before and after surgery. This case suggests that relatively long-term preservation of otolithic function can be achieved following triple semicircular canal occlusion, highlighting its potential as an alternative treatment for patients with Ménière disease.

## Introduction

Ménière disease (MD) is a common, chronic inner ear disease with an incidence of 15–50 per 100,000 population, and is characterized by episodes of spontaneous vertigo, fluctuating sensorineural hearing loss, tinnitus, and aural fullness (1, 2). Since its definitive pathogenesis remains unknown, there is currently no cure for MD. Operative measures such as endolymphatic sac surgery, vestibular neurectomy, and labyrinthectomy are considered when medical treatment fails to relieve vertigo (1). As a conservative procedure, endolymphatic sac surgery was often performed in patients with intractable MD, but the success rate was only 43–84% (3, 4). In 1990, semicircular canal occlusion was successfully applied for the treatment of intractable benign paroxysmal positional vertigo for the first time (5, 6). Owing to this success, some scholars began to discuss the application of triple semicircular canal occlusion (TSCO) surgery for MD (7, 8). Relevant evidence has indicated that TSCO can successfully treat vertigo and preserve hearing in patients with MD (7). However, it is critical to ascertain whether otolithic function can be preserved after TSCO. Therefore, the present study aimed to evaluate whether TSCO surgery can alleviate symptoms of vertigo in patients with MD, and whether otolithic function is preserved after surgery.

## Case Report

### Patient

A 30-year-old woman began experiencing vertigo, hearing loss, tinnitus, and aural fullness in her right ear in December 2011. Based on her medical history, symptoms, and audiometric test results, she was diagnosed with definite MD according to the diagnostic criteria of the American Academy of Head and Neck Surgery (9). Initially, she was administered standardized conservative treatment comprised of betahistine, sodium aescinate, and intratympanic injection of dexamethasone for a period of one year. However, these treatments could not control the vertigo, which continued to recur, sometimes daily. Hence, in January 2014, she was referred to our hospital where she underwent endolymphatic sac decompression of the right ear on March 27, 2014 (shown in Supplementary Figure 1A). Although the vertigo was initially alleviated by the surgery, it recurred 5 months postoperatively with ~5 episodes per month. Thus, the patient was unable to work, continued to feel anxious, and had a strong desire for another surgical treatment to relieve the vertigo. After being informed of the TSCO surgical method, treatment effect, prognosis, and possible related complications in detail, the patient underwent TSCO on September 28, 2014.

### TSCO Procedure

The surgery was performed under general anesthesia via a post-auricular approach. The three semicircular canals were identified and skeletonized (shown in Supplementary Figure 1B). TSCO was then performed as follows: A fenestration of about 2 mm was made in the central portion of the bony canal, as far away as possible from the ampulla, using a 1-mm drill and a hook to pry off the bone, without opening the endosteum or the membranous labyrinth. Great care was taken to avoid injuring the membranous labyrinth as it can lead to serious hearing loss (10). The temporalis fascia, bone dust collected during the procedure, and fibrinogen glue were inserted through the fenestra to compress the endosteum and membranous labyrinth against the bony back wall. The fenestration and surrounding bone were covered with a piece of temporalis fascia and fibrinogen glue. Subsequently, the incision was closed, and the procedure was completed.

### Auditory and Vestibular Function Tests

Pure tone audiometry (Otometrics Conera Audiometer, Otometrics, Taastrup, Denmark) was used to evaluate hearing loss. The caloric test (ICS Aircal, Otometrics) was used to assess lateral semicircular canal function. The cervical vestibular evoked myogenic potential (cVEMP) (Chart EP 2000, Otometrics) was used to evaluate saccular and inferior vestibular pathway functions, while the ocular vestibular evoked myogenic potentials (oVEMP) was used to evaluate the functions of the utricular and superior vestibular pathways (11, 12). The head-impulse paradigm (HIMP) (ICS Impulse, Otometrics) and suppression head-impulse paradigm (SHIMP) were used to evaluate semicircular canal function through the gain of vestibulo-ocular reflex (VOR) (13).

## Outcomes and Follow-Up

### Evaluation of Vertigo and Coexisting Symptoms

One month after the surgery, the patient experienced vertigo; therefore, she was advised bed rest. For the next 6 months, though she experienced occasional dizziness when walking or getting up too quickly, her daily life, and work were unaffected. No hearing deterioration occurred, and her aural fullness improved. The patient recovered completely and did not experience vertigo during the 24 months of follow-up after TSCO surgery (class A) (9). During the entire postoperative period, which lasted until January 2021, the patient experienced vertigo only once in July 2017 (33 months after TSCO surgery), lasting 5 h, which was accompanied by nausea but not vomiting. The intensity of this episode was markedly lower than that of her preoperative episodes. We provided education regarding changing and maintaining a reasonable diet and lifestyle, including reducing sodium, caffeine, alcohol intake, as well as how to manage work-related stress. A combination of oral betahistine and sodium aescinate was administered for 2 weeks and vertigo did not recur. The patient experienced ear fullness when she did not have sufficient rest or was overworked. Moreover, she had already adapted to the continuous tinnitus and it did not influence her life. Further, the severity of tinnitus decreased substantially when she wore hearing aids.

### Auditory and Vestibular Function Evaluation

**Figure 1:**
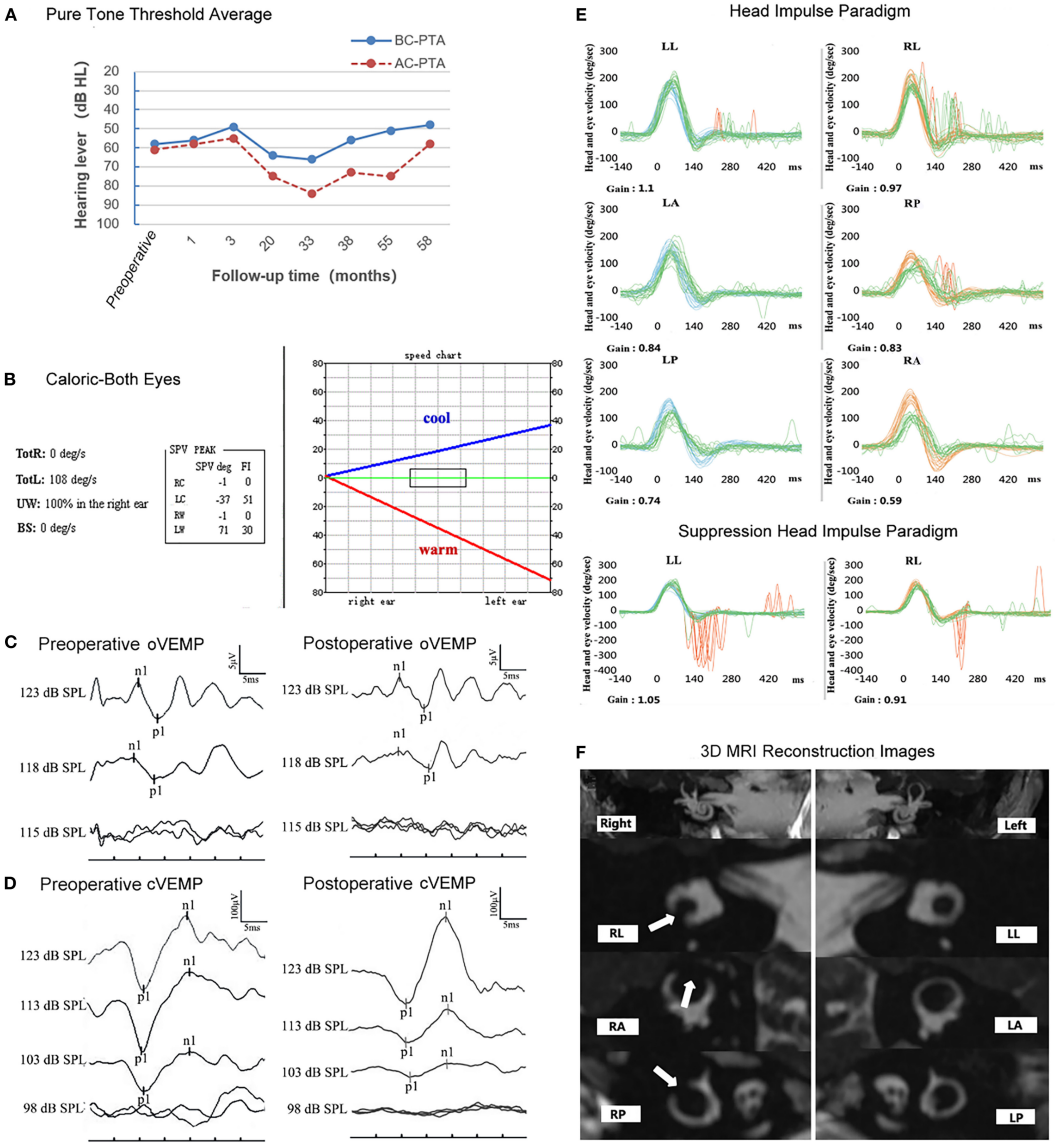
Results of the auditory and vestibular function tests before and after surgery. **(A)** No change in hearing threshold levels before and 58-months after the operation. **(B)** The post-operative caloric test revealed that, regardless of cold or heat stimulation, there was no response in the right horizontal semicircular canal. RC, right cool; LC, left cool; RW, right warm; LW, left warm; UW, unilateral weakness; BS, baseline shift; GA, gain asymmetry; FI, fixation index. **(C,D)** Both the preoperative and postoperative VEMPs could be elicited in the right ear. VEMP, vestibular evoked myogenic potentials; oVEMP, ocular VEMP; cVEMP, cervical VEMP. **(E)** The HIMP gain values for the RL and RP canal were within the normal range. The gain value of the RA canal was significantly lower than that of the LA canal. The SHIMP gain value for the RL canal was within the normal range. HIMP, head-impulse paradigm; SHIMP, suppression head-impulse paradigm; LL, left lateral; LA, left anterior; LP, left posterior; RL, right lateral; RA, right anterior; RP, right posterior. **(F)** Post-operative 3D-MRI reconstruction images of the semicircular canal revealing three completely blocked semicircular canals in the right ear (as indicated by the white arrow).

A series of auditory and vestibular tests were performed to evaluate her hearing and otolithic functions before and after surgery (Figure 1). There was no significant increase in the pure tone average threshold at 58 months after surgery (Figure 1A). The right lateral semicircular canal did not respond to the caloric test (Figure 1B), indicating that the lateral semicircular canal was obstructed successfully. The oVEMP and cVEMP could be elicited both preoperatively and postoperatively (Figures 1C,D). The follow-up caloric test results and VEMPs at different postoperative stages are shown in Supplementary Figures 2, 3. The HIMP data at 2 months postoperative showed that the gains of the right three semicircular canals were lower than that of the left after TSCO. These data showed that the occlusion was successful. The HIMP test at 58 months after surgery demonstrated that the gain of the right anterior semicircular canal was lower than that of the left anterior semicircular canal (Figure 1E). The HIMP gain values for the right lateral semicircular canal and posterior semicircular canal were within the normal range. At 76 months postoperatively, the gain values of all three semicircular canals were within the normal range; however, saccades continued to occur (shown in Supplementary Figure 4). The SHIMP gain value for the right lateral semicircular canal was within the normal range (Figure 1E). The postoperative magnetic resonance imaging reconstruction showed that the three semicircular canals of the right ear were completely blocked (Figure 1F).

## Discussion

While various surgical procedures including endolymphatic sac decompression, labyrinthectomy, and vestibular neurotomy are available for patients with MD, the best choice remains controversial, as each method has its advantages and disadvantages (4). In endolymphatic sac decompression surgery, vertigo relief is observed in ~72.2% of patients (14). Although labyrinthectomy yields a higher success rate in this respect, the procedure can lead to severe hearing loss. Vestibular neurotomy is associated with a high surgical risk and may lead to life-threatening complications (15). In contrast, a previous study using a cat model demonstrated that TSCO can effectively treat vertigo, protect hearing, and preserve otolith function (15). In accordance with this finding, the present case suggests that TSCO is safe and effective for treating MD in human patients.

Rotational vertigo is the major concern in most patients with MD. The sensation of rotational vertigo is linked to stimuli in the cupula of the semicircular canals and is caused by excess liquid in the endolymphatic space and the mixture of endolymph and perilymph. Surgical occlusion of the canals reduces stimulation of the cupula due to endolymph flow or the mixture of fluids in the membranous semicircular canal, eliminating the occurrence of vertigo episodes (7). Further, the functions of the semicircular canal are selectively eliminated, meaning that it is no longer affected by mechanical, location-based, or chemical factors; however, quick and complete restoration of vestibular function can be observed postoperatively (15). Our patient experienced only one post-operative vertigo episode 33 months after TSCO. We hypothesized that TSCO changes the microenvironment of the inner ear, interrupting the periodic pathological cycle of MD (onset-intermittent-onset). Therefore, beyond the mechanism of mechanical obstruction, further studies are required to explore the complex pathophysiological mechanisms of this phenomenon. Notably, TSCO can cause hearing loss of up to 30%, which may be associated with labyrinthitis and perilymph fistulae (10, 16).

In the current patient, the oVEMP and cVEMP were preserved both preoperatively and at 76 months postoperatively, suggesting that otolithic function was retained after TSCO. The preservation of VEMPs is conducive to vestibular rehabilitation and is especially suitable for patients with bilateral MD. At the same time, the results of the caloric test suggested that the right semicircular canal was paralyzed, indicating successful occlusion of the lateral semicircular canal. Furthermore, the HIMP gain values for the right three semicircular canals were lower than that of the left three semicircular canals at the early stage after TSCO; however, the gains of the right canals gradually recovered to values within the normal range, suggesting that damage to the hair cells of the ampulla was minimal after TSCO, and that vestibular compensation had been established. However, our HIMP results were inconsistent with those of some previous studies that reported a permanent decrease in VOR gain to head impulses after canal occlusion in humans (17, 18). Nonetheless, our data are consistent with those of other studies in which the VOR gain returned to normal postoperatively (19, 20). Therefore, further studies are required to determine the mechanism by which video head-impulse test results gradually return to normal following surgery.

## Conclusion

We performed TSCO in a patient with definite MD to determine whether the procedure represents a safer and simpler alternative for the treatment of intractable vertigo. Clinical testing indicated that VEMPs were preserved during the 76-month follow-up period. Therefore, we hypothesized that TSCO can effectively eliminate MD-related vertigo while preserving otolith function. The current case highlights the potential of TSCO as an alternative treatment for intractable MD.

## Data Availability Statement

The original contributions presented in the study are included in the article/Supplementary Material, further inquiries can be directed to the corresponding author/s.

## Author Contributions

QZ and YC were the surgeons in this case. YZ and YC wrote the manuscript. ZC and FC completed data acquisition. QZ reviewed and edited the manuscript. All authors contributed to the article and approved the submitted version.

## Funding

This study was supported by the National Natural Science Foundation of China (Grant Nos. 81970891 and 82171137) and the Shaanxi Major International Cooperative Project of China (Grant No. 2020KWZ-019).

## Conflict of Interest

The authors declare that the research was conducted in the absence of any commercial or financial relationships that could be construed as a potential conflict of interest.

## Publisher's Note

All claims expressed in this article are solely those of the authors and do not necessarily represent those of their affiliated organizations, or those of the publisher, the editors and the reviewers. Any product that may be evaluated in this article, or claim that may be made by its manufacturer, is not guaranteed or endorsed by the publisher.
