# Supplementary material for: Case Report: Preservation of Otolithic Function After Triple Semicircular Canal Occlusion in a Patient With Intractable Ménière Disease
Source: Front Neurol. 2021 Dec 23;12:713275. doi: 10.3389/fneur.2021.713275 (PMC8732767; doi:10.3389/fneur.2021.713275)
Supplement: Supplementary file 1 [file Data_Sheet_1.docx]

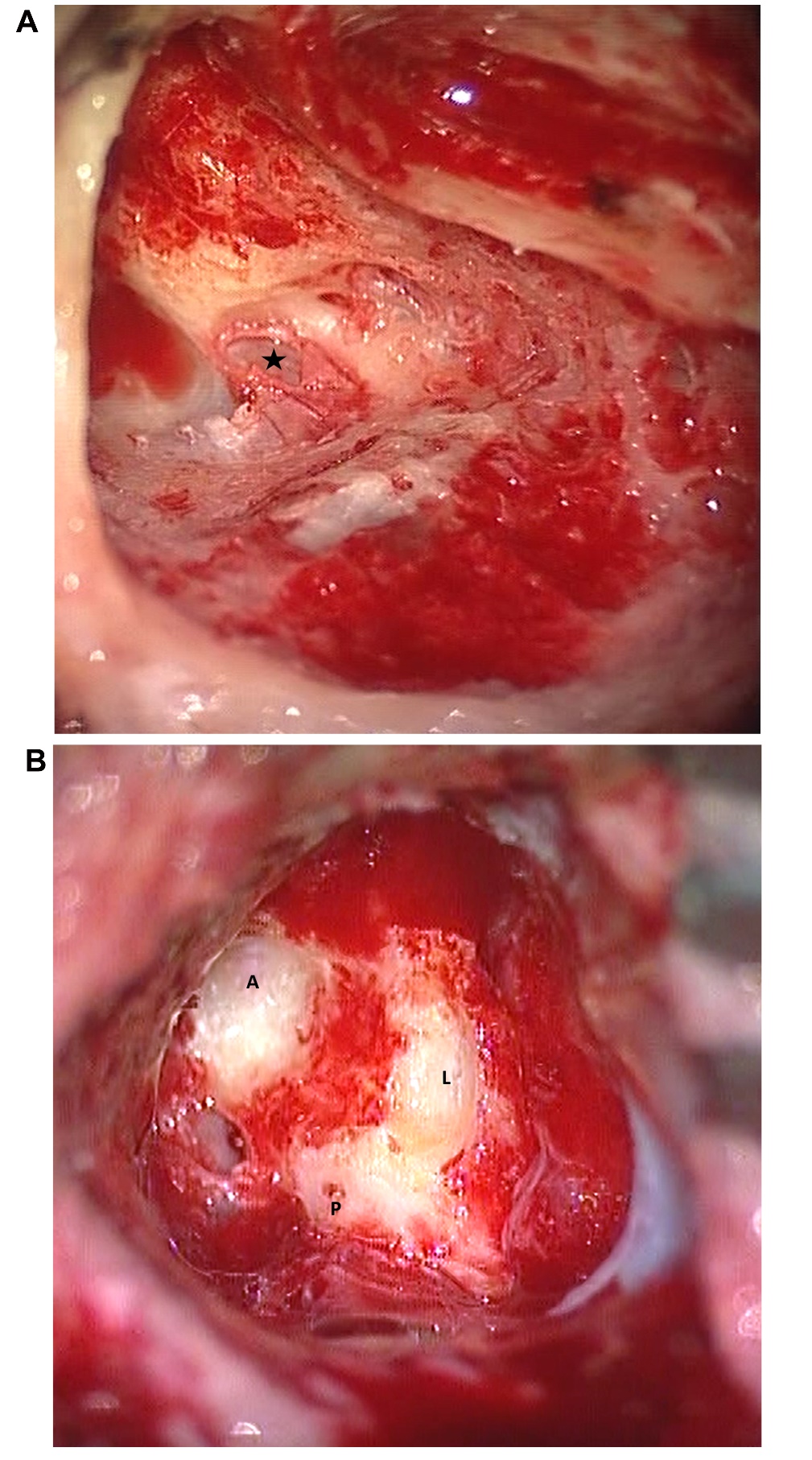


**Figure 1** **Surgery pictures. A** The picture of endolymphatic sac decompression surgery. The black five-pointed star represents the endolymphatic sac that has been incised. **B** Three semicircular canals after occluded. A: anterior semicircular canal; L: lateral semicircular canal; P: posterior semicircular canal.


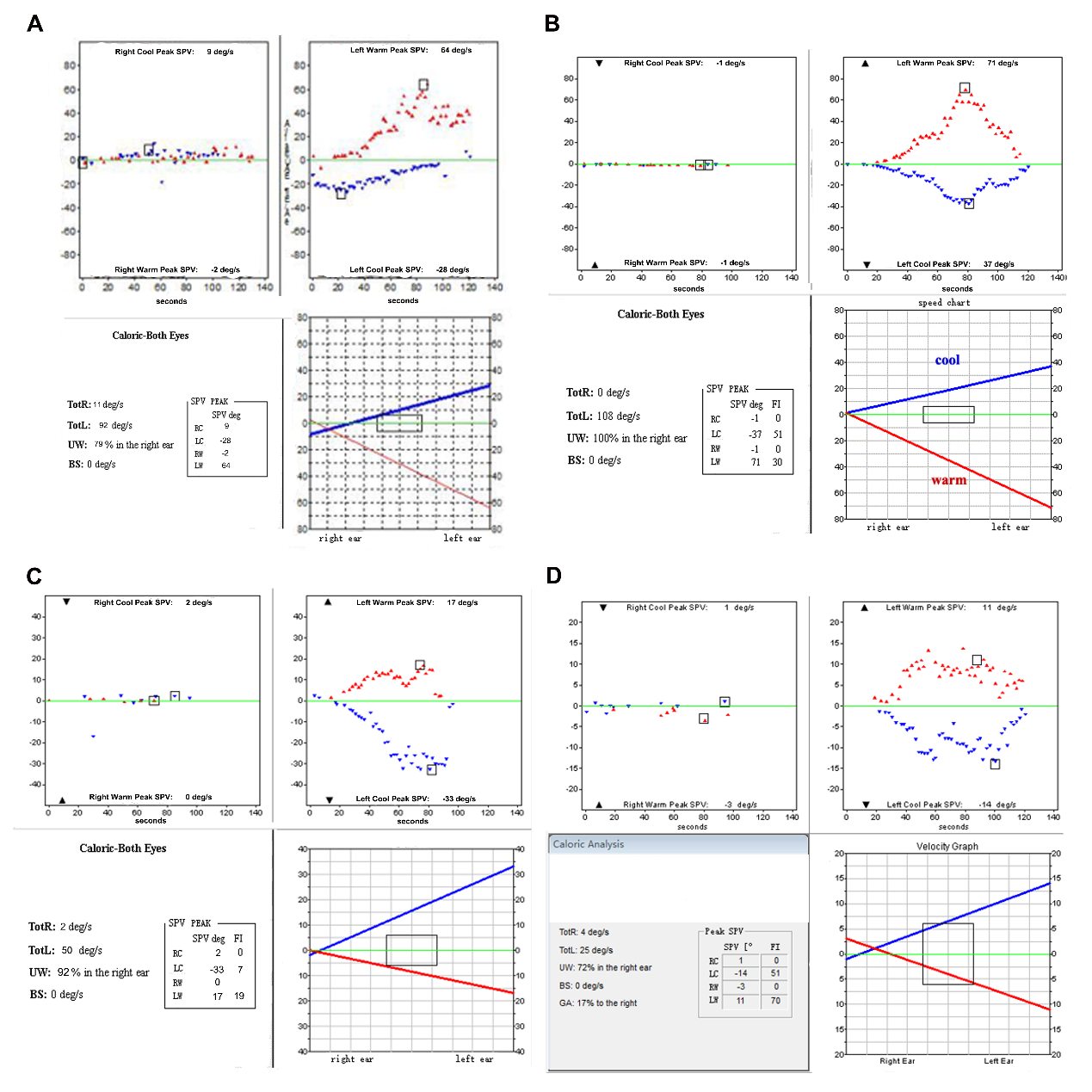


**Figure 2** **Caloric test after TSCO**. **A** Caloric test of 20 months after TSCO. **B** Caloric test of 38 months after TSCO. **C** Caloric test of 55 months after TSCO. **D** Caloric test of 76 months after TSCO.


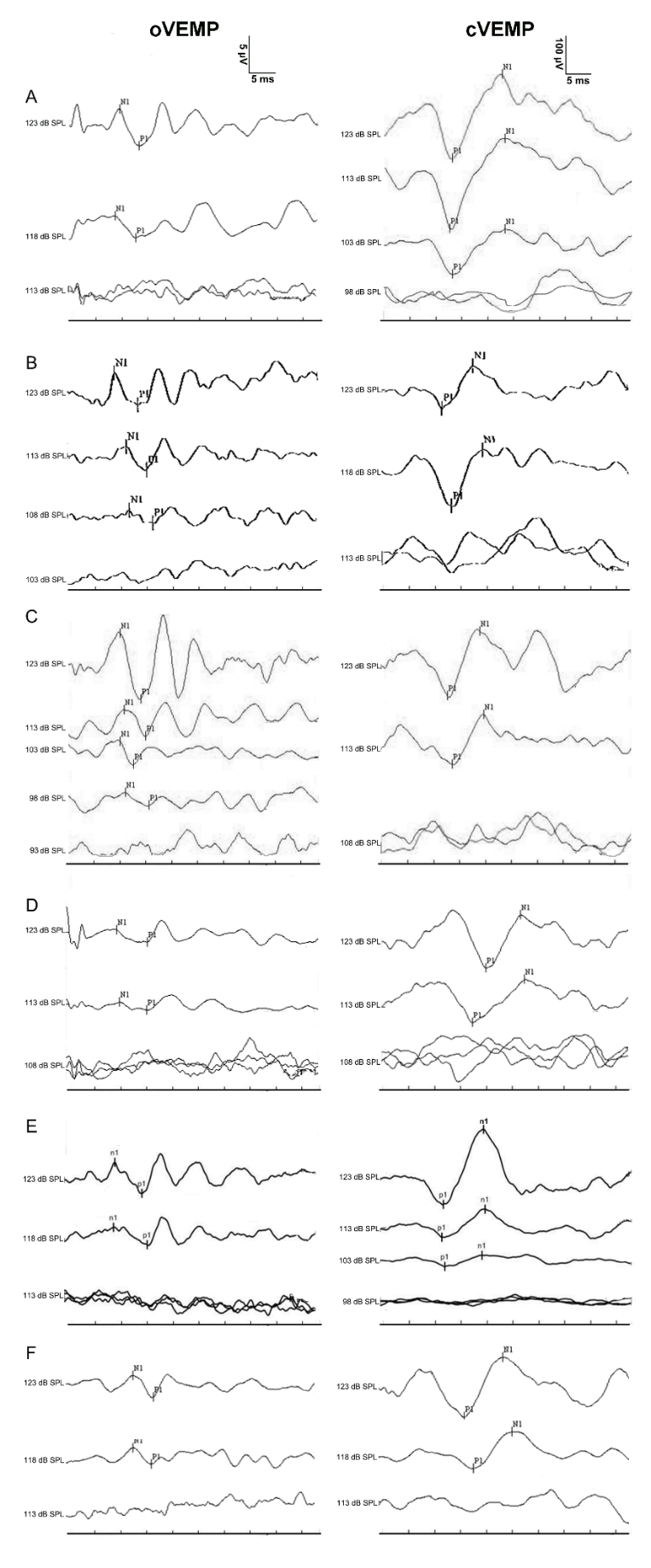


**Figure 3** **VEMPs test before and after TSCO. A** Preoperative VEMPs test. **B** VEMPs test of one month after TSCO. **C** VEMPs test of 33 months after TSCO. **D** VEMPs test of 38 months after TSCO. **E** VEMPs test of 58 months after TSCO. **F** VEMPs test of 76 months after TSCO.


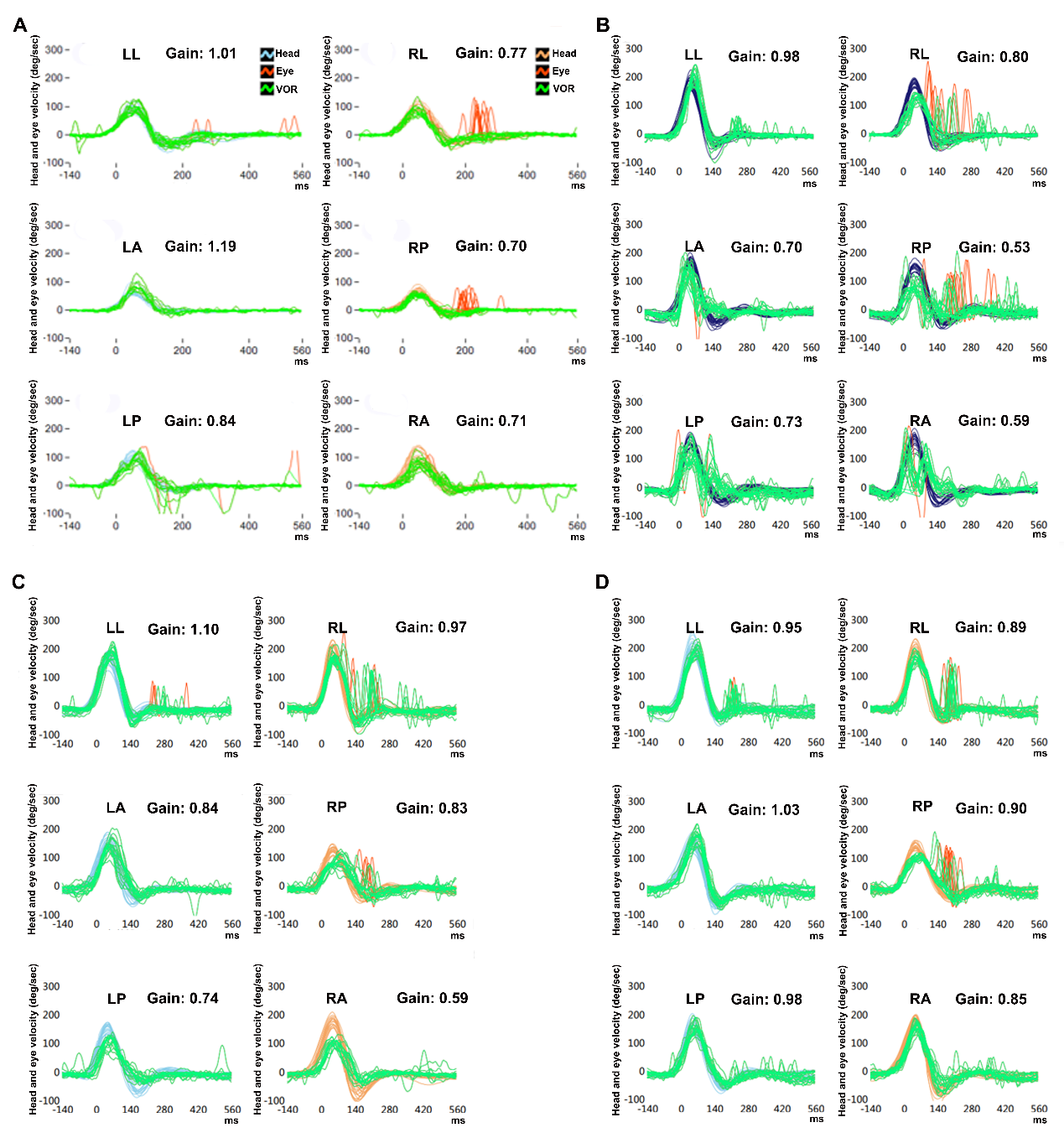


**Figure 4** **HIMP test after TSCO.** **A** HIMP test of 3 months after TSCO. **B** HIMP test of 38 months after TSCO. **C** HIMP test of 58 months after TSCO. **D** HIMP test of 76 months after TSCO. LL: left lateral; LA: left anterior; LP: left posterior; RL: right lateral; RA: right anterior; RP: right posterior.
